# Evaluating Fluorinated-Aniline Units with Functionalized Spiro[Fluorene-9,9′-Xanthene] as Hole-Transporting Materials in Perovskite Solar Cells and Light-Emitting Diodes

**DOI:** 10.3390/nano14121044

**Published:** 2024-06-18

**Authors:** Kuo Liu, Liang Sun, Qing-Lin Liu, Bao-Yi Ren, Run-Da Guo, Lei Wang, Ya-Guang Sun, You-Sheng Wang

**Affiliations:** 1 Key Laboratory of Inorganic Molecule-Based Chemistry of Liaoning Province, College of Science, Shenyang University of Chemical Technology, Shenyang 110142, China; 15233487432@163.com (K.L.); lql1347585318@163.com (Q.-L.L.); sunyaguang@syuct.edu.cn (Y.-G.S.); 2 Wuhan National Laboratory for Optoelectronics, Huazhong University of Science and Technology, Wuhan 430074, China; lsiuang@hotmail.com (L.S.); wanglei@mail.hust.edu.cn (L.W.); 3 Institute of New Energy Technology, College of Physics & Optoelectronic Engineering, Jinan University, Guangzhou 510632, China

**Keywords:** spiro[fluorene-9,9′-xanthene], substitution effect, hole-transporting materials, perovskite optoelectronic devices

## Abstract

In the field of perovskite optoelectronics, developing hole-transporting materials (HTMs) on the spiro[fluorene-9,9′-xanthene] (SFX) platform is one of the current research focuses. The SFX inherits the merits of spirobifluorene in terms of the configuration and property, but it is more easily derivatized and regulated by virtue of its binary structure. In this work, we design and synthesize four isomeric SFX-based HTMs, namely ***m*-SFX-*m*F**, ***p*-SFX-*m*F**, ***m*-SFX-*o*F**, and ***p*-SFX-*o*F**, through varying the positions of fluorination on the peripheral aniline units and their substitutions on the SFX core, and the optoelectronic performance of the resulting HTMs is evaluated in both perovskite solar cells (PSCs) and light-emitting diodes (PeLEDs) by the vacuum thermal evaporating hole-transporting layers (HTLs). The HTM ***p*-SFX-*o*F** exhibits an improved power conversion efficiency of 15.21% in an inverted PSC using CH_3_NH_3_PbI_3_ as an absorber, benefiting from the deep HOMO level and good HTL/perovskite interface contact. Meanwhile, the HTM ***m*-SFX-*m*F** provides a maximum external quantum efficiency of 3.15% in CsPb(Br/Cl)_3_-based PeLEDs, which is attributed to its perched HOMO level and shrunken band-gap for facilitating charge carrier injection and then exciton combination. Through elucidating the synergistic position effect of fluorination on aniline units and their substitutions on the SFX core, this work lays the foundation for developing low-cost and efficient HTMs in the future.

## 1. Introduction

In the last decade, halide perovskite semiconductors have risen sharply in the field of advanced optoelectronic devices such as solar cells and light-emitting diodes, thanks to their comprehensive advantages in terms of the cost and performance [1]. For perovskite solar cells (PSCs), the power conversion efficiency (PCE) has approached 25% in a single-junction device using inorganic–organic hybrid halide perovskite materials as light absorbers [2]. Meanwhile, the fully inorganic cesium lead halide perovskites with the characteristics of a tunable emission color, high photoluminescent quantum yield as well as narrow emission line widths [3], have been considered ideal candidates for light-emitting diode applications, so-called PeLEDs [4,5]. Given the intrinsic semiconductor property of halide perovskite materials, the corresponding optoelectronic devices basically employ planar heterojunction architectures of the *n*-*i*-*p* or *p*-*i*-*n* form, in which the *n*- or *p*-type material is used to transport photo-generated or electro-injected charge carriers.

Besides the most important photoactive layer being based on perovskite materials [6,7], the hole-transporting materials (HTMs) play a key role in both improving the PCE and enhancing the stability of PSCs with either regular or inverted architectures [8,9], such as the well-known milestone molecule Spiro-OMeTAD [10]. The successful case benefits from integrating aniline units with excellent hole-transporting capability onto a three-dimensional (3D) spiro backbone. When the symmetric spirobifluorene (SBF) core is tetra-functionalized with aniline units, the pseudo-globular structure can provide high amorphous stability and spatial homogeneity in a solid-state film by virtue of its large molecular weight and small intermolecular cohesion [11]. Hence, a great deal of research works based on spiro compounds have been conducted to develop high-performance HTMs. By refining the research results, it can be found that spiro[fluorene-9,9′-xanthene] (SFX) is an ideal alternative to SBF, as the asymmetric spiro structure of SFX makes its derivatization and regulation more flexible than SBF [12,13,14]. For the electroactive units of HTMs, the electron-rich aniline-like segments are preferred because of their suitable properties, for instance, the lone pair of electrons of nitrogen atoms facilitate the formation of radical cation intermediates [15]. Furthermore, the methoxy side group extends the frontier molecular orbitals (FMOs) of spiro-OMeTAD, leading to a large FMO overlap between adjacent molecules and then enhancing the hole-transporting capability [16]. In light of this, the modification of the aniline unit becomes a significant topic [17]. The fluorine atom has nearly the same size as the hydrogen atom; therefore, there is a negligible influence on the molecular configuration of HTMs as fluorination is carried out on the aniline units. On another scale, the strong induced dipole along the C−F bond can lower the FMOs’ energy levels and regulate the interface interaction between the hole-transporting layer (HTL) and the light absorption layer. Based on Spiro-OMeTAD, Yang and co-workers introduced one fluorine atom to the *ortho*-(*o*F) or *meta*-position (*m*F) of the methoxy group of the aniline unit, denoted as **Spiro-*o*F** and **Spiro-*m*F**, where the PSC device using **Spiro-*o*F** as the HTM displays a PCE as high as 24.8% and outstanding stability that is superior to Spiro-OMeTAD-based devices [18].

For the PeLED devices, organic small molecular HTMs are also desired because this class of HTMs can be directly evaporated on indium–tin oxide (ITO) anodes to form high-quality HTLs [19]. Such an architecture in a PSC is a so-called inverted device that usually possesses better stability than the *n*-*i*-*p*-form PSCs [20]. In addition, the dopants that are employed for increasing the mobility of HTMs are barely used in the thermal evaporation process. As a result, the dopant-free HTLs are practicable and stable for perovskite optoelectronic devices [21,22].

In this context, we report four isomeric HTMs termed ***m*-SFX-*m*F**, ***p*-SFX-*m*F**, ***m*-SFX-*o*F**, and ***p*-SFX-*o*F** on the basis of our previous work [23,24], which are also inspired by the fluorination strategy of aniline units [18]. The performances of the resulting HTMs are evaluated in both PSC and PeLED devices. The *o*F-unit-modified HTMs present better performance for the inverted PSCs with thermally evaporated dopant-free HTLs, especially for the ***p*-SFX-*o*F** in which the 3′- and 6′-positions of the xanthene moiety of SFX are aminated beside the fluorene moiety, where the corresponding PSC offers a PCE of 15.21%. Interestingly, in the evaporated fully inorganic perovskite-based PeLEDs, the device exhibits 3.15% external quantum efficiency (EQE) when the *m*F-units are introduced at the 2-, 2′-, 7- and 7′-positions of the SFX. Thus, the synergistic position effect of the fluorine atom on the aniline units and their substitutions on the SFX core is clarified for PSC and PeLED applications, respectively.

## 2. Materials and Methods

### 2.1. General Methods

The ^1^H/^13^C NMR spectra were obtained with a Bruker Avance500 II spectrometers (Billerica, MA, USA) at ambient temperature by utilizing deuteron dimethyl sulfoxide (DMSO-*d*_6_) as a solvent and tetramethylsilane (TMS) as a standard. The mass spectra were obtained with a Waters Xevo G2-XS Tof (Milford, MA, USA). The UV–vis absorption spectra were measured using a UV-2550 Spectrophotometer (Shimadzu, Kyoto, Japan). Cyclic voltammetry (CV) was performed on a CH1760E electrochemical workstation with a scan rate of 100 mV s^−1^ at room temperature, using a glassy carbon as the working electrode, a platinum wire as the counter electrode, and Ag/Ag^+^ as the reference electrode, standardized for the redox couple ferrocene/ferricenium. The samples were prepared in DCM solution with 0.1 mol L^−1^ of tetrabutylammonium hexafluorophosphate at a scan rate of 100 mV^−1^ s^−1^ at room temperature. The Ag/Ag+ reference electrode was calibrated using a ferrocene/ferrocenium redox couple as an internal standard whose oxidation potential was set at 5.09 eV with respect to the zero-vacuum level. The HOMO energy levels of the HTMs were obtained from the equation *E*_HOMO_ = −[*E_ox_*_._ − *E*_(Fc/Fc_^+^_)_ + 5.1] eV and the LUMO levels from the equation *E*_LUMO =_ *E*_HOMO_ + *E*_g_^opt^. The thermogravimetric analyses (TGA) were performed using a Shimadzu DTG-60H thermogravimetric analyzer under a heating rate of 10 °C min^−1^ and a nitrogen flow rate of 50 cm^3^ min^−1^. The structures of the perovskite films were analyzed using X-ray diffractometry (XRD) using a D8 Advance (Bruker, Saarbrücken, Germany) diffractometer equipped with Cu *K*_α_ radiation (*λ* = 0.1542 nm). The PL spectrum was measured by Edinburgh Instruments (FLS980, Livingston, UK) with a Picosecond Pulsed UV-LASTER (LASTER377) (Livingston, UK) as the excitation source.

### 2.2. Fabrication of PSCs

The perovskite solar cell device was fabricated by vacuum thermal evaporation of the hole transport layer. The substrate used a transparent conductive glass of 20 Ω/sq ITO (indium–tin oxide) as the anode, which was ultrasonicated for 15 min in an ultrasonic cleaning machine with acetone, anhydrous ethanol and deionized water, respectively. After 10 min of UV–O_3_ treatments, it was steamed in a vacuum chamber with a vacuum degree of less than 1.33 × 10^−7^ Pa. The 20 nm thick neat***m*****-SFX-*m*F**, ***m*-SFX-*o*F**, ***p*-SFX-*m*F** and ***p*-SFX-*o*F** HTLs were evaporated onto the ITO substrate. The other functional layers were fabricated as described in the literature but without the surface passivation [25].

### 2.3. Fabrication of PeLEDs

Our thermal evaporator equipment is a precision system with two vacuum chambers, one for the preparation of perovskite films and the other for the deposition of function layers. The different substrates were transferred into a vacuum chamber for the deposition of perovskite films with 80 nm thickness. Unless otherwise specified, Cs/Pb was chosen and the temperature of the sample holder was set to 20 °C. In the case of a low substrate temperature, the temperature was set to −10 °C. The ratio of PbBr_2_:CsCl:CsBr was chosen as 1:0.85:0.55. After the fabrication of the perovskite films, the films were transferred to the other vacuum chamber. Finally, TPBi (20 nm) and LiF/Al electrodes (1 nm/100 nm) were deposited using a thermal evaporation system through a shadow mask under a high vacuum of ≈4 × 10^−4^ Pa. The active area of the device was 6 (2 × 3) mm^2^. The UV-curable adhesive was used to glue a glass encapsulation sheet over the active area of the device.

## 3. Results and Discussion

### 3.1. Synthesis, Theoretical Calculation, Photophysical and Electrochemical Properties

**Scheme 1:**
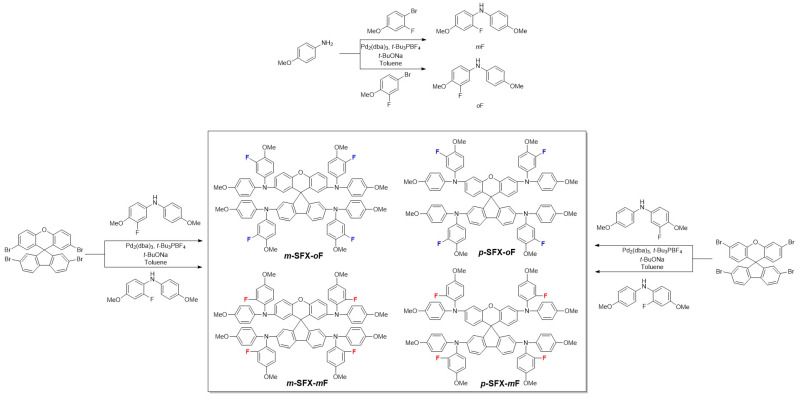
The synthetic routes of ***m*-SFX-*o*F**, ***p*-SFX-*o*F**, ***m*-SFX-*m*F**, and ***p*-SFX-*m*F**.

To develop the novel HTMs, the fluorinated aniline units were smoothly linked to the SFX core through Buchwald–Hartwig aminations, resulting in four molecules, ***m*-SFX-*m*F**, ***p*-SFX-*m*F**, ***m*-SFX-*o*F** and ***p*-SFX-*o*F**, respectively, with satisfactory yields varying from 83% to 91%. The synthetic routes and molecular structures of the HTMs are presented in Scheme 1. The binary spiro-system of the SFX doubles the HTM isomers in comparison with the SBF derivatives. Similar to Spiro-OMeTAD, the designed molecules also have orthometric configurations (Figure S1), thanks to the 3D SFX core. The fluorination effect of the aniline units on the HTMs has been illustrated, where the highest occupied molecular orbital (HOMO) levels are lowered and lead to well-matched energy-level alignment with perovskite for hole extraction and transport [26]. Moreover, fluorination can improve the hydrophobicity of the HTLs and enhance the stability of the devices [27].

**Figure 1:**
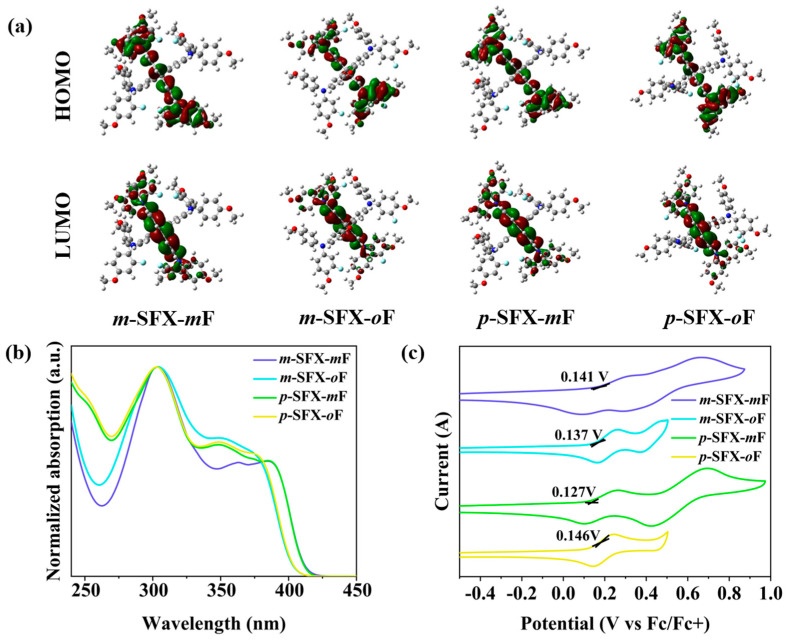
DFT calculation, optical and electrochemical characteristics of ***m*-SFX-*m*F**, ***m*-SFX-*o*F**, ***p*-SFX-*m*F** and ***p*-SFX-*o*F**. (**a**) Electron density distributions of HOMO and LUMO. (**b**) Normalized UV–vis absorption spectra in dilute CH_2_Cl_2_ solutions. (**c**) CV curves in CH_2_Cl_2_ solutions.

For a better understanding of the structure–property relationship of the prepared HTMs, we carried out the simulated calculation using density functional theory (DFT) B3LYP with the 6-31g (d,p) basis set. The molecular configurations show that the dihedral angles between the xanthene and fluorene moiety are all about 90° (Figure 1a and Figure S1), suggesting that the compounds exhibit highly distorted structures. The 3D structures can endow the molecules with good stability and are beneficial for the successful deposition of HTLs by a thermal evaporation process. The spatial distributions of the frontier molecular orbitals (FMOs) of the HTMs are depicted in Figure 1a, where the HOMOs mainly spread over the triphenylamine segments of the fluorene moiety, while the lowest unoccupied molecular orbitals (LUMOs) tend to gravitate toward the core. According to the results of the theoretical calculation, it can be found that the HOMO level is governed by the relative position between the fluorine atom and the methoxyl group. When the fluorine atom locates on the *ortho*-position of the methoxyl group of aniline, that is, the *o*F unit, the corresponding ***m*-SFX-*o*F** and ***p*-SFX-*o*F** possess more a stabilized HOMO level than the ***m*-SFX-*m*F** and ***p*-SFX-*m*F** in which the fluorine atom lies on the *meta*-position of the methoxyl groups. This is attributed to the stronger electron-withdrawing effect of the *ortho*-substituted fluorine atom to the methoxyl groups relative to the *meta*-substituted fluorine atom. The estimated HOMO levels of ***m*-SFX-*m*F**, ***m*-SFX-*o*F, *p*-SFX-*m*F** and ***p*-SFX-*o*F** are −4.20, −4.32, −4.19 and −4.37 eV, respectively [18]. These results suggest that the fluorinated HTMs with lowered HOMO levels would promote the hole extraction and transport in perovskite-based optoelectronic devices [28,29].

The absorption spectra of the HTMs were measured in dilute dichloromethane (10^−5^ M), as shown in Figure 1b, all with two absorption bands. The absorption band around 270–325 nm is attributed to the n→π* of SFX; thus, there is no significant difference in the bands for the four molecules. The broad absorption band around 325–400 nm is assigned to the π→π* transition of the molecular conjugate skeletons. In this band, the absorption of ***m*-SFX-*m*F** and ***p*-SFX-*m*F** shows obvious redshift compared with ***m*-SFX-*o*F** and ***p*-SFX-*o*F**. According to the absorption edges, the optical bandgaps of ***m*-SFX-*m*F**, ***m*-SFX-*o*F**, ***p*-SFX-*m*F** and ***p*-SFX-*o*F** are estimated to be 2.99, 3.06, 2.99 and 3.05 eV, respectively. The photoluminescent spectra were also measured, as shown in Figure S2. The emission peaks of ***m*-SFX-*m*F** and ***p*-SFX-*m*F** are both located at 423 nm, while ***m*-SFX-*o*F** and ***p*-SFX-*o*F** present a blueshift of 10 nm relatively.

Cyclic voltammetry (CV) tests were performed to determine the HOMO levels of the HTMs. According to the CV curves (Figure 1c), the first oxidation potentials of ***m*-SFX-*m*F**, ***m*-SFX-*o*F, *p*-SFX-*m*F**, and ***p*-SFX-*o*F** are 0.141, 0.137, 0.127 and 0.146 V, respectively, and then the HOMO levels are deduced to be −5.24, −5.24, −5.23 and −5.25 eV from the formula −e [5.1 eV + *E*ox onset (vs. *E*_Fc/Fc+_)], respectively. The valence band (VB) of perovskite materials is located about −5.4~−6.1 eV [29], which is matched with the HOMO levels of our molecules; hence, the hole extraction or injection is easy to conduct when using the molecules as HTLs in perovskite optoelectronic devices. Indeed, the deep HOMO level can not only enhance the *V*_oc_ for PSCs but can also reduce the operating voltage of PeLEDs. The thermal properties of the HTMs were studied by thermogravimetric analysis (TGA) and differential scanning calorimetry (DSC), and the curves are shown in Figure S3. The HTMs all exhibit excellent thermal stability with a decomposition temperature (*T*_d_) above 395 °C, and the glass transitions of the HTMs are not observed in the DSC measurements. These results imply that the HTMs possess low crystallinity and good morphological stability in the solid state. The properties of the HTMs are summarized in Table 1.

### 3.2. PSC Performance

**Figure 2:**
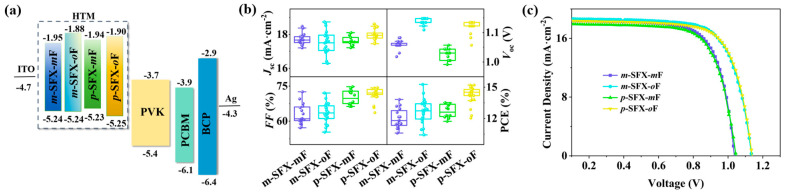
Photovoltaic performance. (**a**) Molecular energy level alignments of the HTMs. (**b**) PCE distribution of each HTM. For each type of PSC, the solid transverse lines in the boxes are the average values, and the error bars show the highest and lowest values at each point. (**c**) *J*-*V* curves of the devices with a maximum PCE.

To evaluate the photoelectronic performance of the HTMs, we initially fabricated inverted PSC devices with the configuration (Figure 2a) of ITO/HTMs/CH_3_NH_3_PbI_3_/PCBM/bathocuproine (BCP)/Ag, using the prepared molecules as dopant-free HTLs, by means of vacuum evaporation. The thicknesses of the HTLs were adjusted to optimize the PSC architectures. The measured *J-V* curves and performance parameters of the fabricated PSCs with different HTL thicknesses are shown in Figures S4–S7 and summarized in Table S1. According to these results, finally, we select the 20 nm thickness HTLs to evaluate the performance of the HTMs. The parameters, including the short-circuit current density (*J*_sc_), open-circuit voltage (*V*_oc_) as well as fill factor (*FF*), and the PCE distributions of the evaluated devices points are presented in Figure 2b. The ***m*-SFX-*o*F**- and ***p*-SFX-*o*F**-based PSCs show higher *J*_sc_ than the other two devices, implying that the *o*F-unit-functionalized molecules possess distinguished hole-transporting capability as well as good electrical contact with the perovskite absorber [30]. It has been found that minimizing the energetic offset between HTLs and the perovskite active layer can endow PSCs with high *V*_oc_ values and reduce the non-radiative recombination losses [31,32]. We speculate that the *o*F-units induced a stronger interface interaction due to the fluorine atom swinging outward; however, this effect is absent for the *m*F-units with an inward fluorine atom [14]. Therefore, the *o*F-unit-substituted HTMs, ***m*-SFX-*o*F** and ***p*-SFX-*o*F**, likewise exhibit superior values, attributed to their relatively deep HOMO levels. However, functionalizing the 2-, 3′-, 6′-, and 7-positions of the xanthene moiety with both *m*F-units and *o*F-units helps with the improvement of the *FF* (Figure 2b). Based on abovementioned parameters, the statistic PSC performance points present the average PCEs of 11.68%, 12.70%, 12.59%, and 14.29% for ***m*-SFX-*m*F**, ***m*-SFX-*o*F**, ***p*-SFX-*m*F** and ***p*-SFX-*o*F**, respectively, meaning that ***p*-SFX** designs exhibit better performance, especially for oF-unit functionalization. Comparing the PCEs of the PSCs, the ***p*-SFX-*o*F**-based PSCs possess the best performance among the isomeric HTMs in terms of both the average and the maximum PCEs. Figure 2c shows the *J*-*V* curves derived from the devices with the maximum PCEs. The higher *V*_oc_ of the ***m*-SFX-*o*F**- and ***p*-SFX-*o*F**-based PSCs illustrates the role of the *o*F-unit in influencing the HOMO levels. The best performance of the ***p*-SFX-*o*F**-based device with the maximum and average PCEs of 15.21% and 14.29%, respectively, manifests the synergistic position effect of fluorination on aniline units and their substitutions on the SFX core (Table 2).

**Figure 3:**
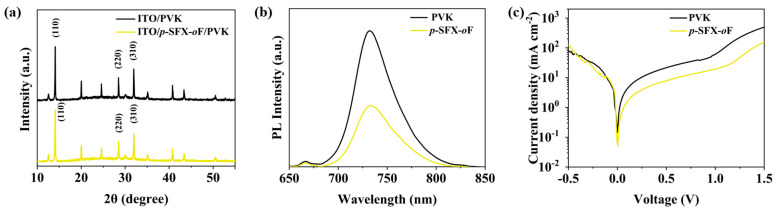
(**a**) XRD patterns; (**b**) PL; and (**c**) *J-V* curves under dark conditions of neat perovskite film and with the ***p*-SFX-*o*F** sample.

The X-ray diffractions (XRDs) and steady-state photoluminescence (PL) of the neat CH_3_NH_3_PbI_3_ film and ***p*-SFX-*o*F**-attended sample were measured to investigate the perovskite/HTM interface interaction and hole extraction effect. The XRD pattern of the neat CH_3_NH_3_PbI_3_ presents strong diffraction peaks at 14.08°, 28.51°and 31.97°, corresponding to the (110), (220) and (310) crystal planes (Figure 3a), respectively, confirming the exquisite and ordered crystalline perovskite film. For the ***p*-SFX-*o*F**-attended sample, the diffraction pattern is almost perfectly consistent with the neat CH_3_NH_3_PbI_3_ film, revealing that the construction of the CH_3_NH_3_PbI_3_ layer is unaffected by the HTL. In Figure 3b, the emission intensity of the ***p*-SFX-*o*F**-contained sample at 732 nm decreases by 55% relative to the neat CH_3_NH_3_PbI_3_ film. This variation demonstrates that the charge carriers finish an effective transfer at the perovskite/HTL interface. Figure 3c shows the semilogarithmic plot of the *J*−*V* curves under dark conditions for confirming the inhibition effects of ***p*-SFX-*o*F** on the leakage and surface recombination [33]. For a conventional *p*-*n* junction, the relationship between the reverse bias current density (*J*_o_) and the *V*_oc_ follows the equation,

(1) $$V_{oc}=\left(\frac{nk_{B}T}{q}\right)\ln\left(\frac{J_{ph}}{J_{o}}+1\right)$$

where *J*_ph_ is the photocurrent density, *q* is the charge of one electron, *n* is the ideality factor, *k*_B_ is the Boltzmann constant, and *T* is the absolute temperature. The current density of the device with the ***p*-SFX-*o*F** HTL under reverse bias is lower than that of the reference device, which means that the charge carrier recombination inside the PSC is restrained and leads to the improvement of the PCE because of a lower *J*_o_ resulting in a larger *V*_oc_ according to Equation (1) [34,35].

### 3.3. PeLED Performance

**Figure 4:**
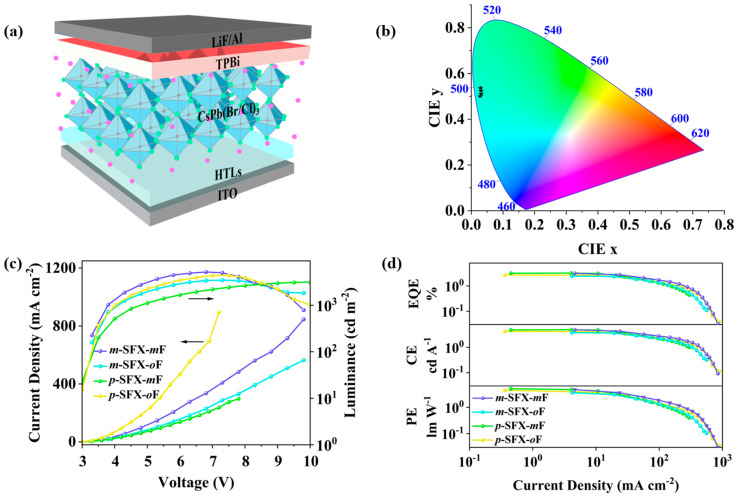
PeLED performance. (**a**) Device architecture; (**b**) CIE (0.55, 0.53~0.56) chromaticity diagram recorded under an operating voltage of 6.0 V; (**c**) *J-V-L* curves; and (**d**) EQE-, PE- and CE-current density.

Considering the diversity and adjustability of the series of HTMs, as well as the attractive potential of fully inorganic halide perovskite for electroluminescent application, we further fabricated PeLED devices through a complete evaporation process. The PeLED devices adopt the following architecture: ITO/HTLs (30 nm)/CsPb(Br/Cl)_3_ (100 nm)/1,3,5-Tris(1-phenyl-1*H*-benzimidazol-2-yl)benzene (TPBi, 20 nm)/LiF (1 nm)/Al (100 nm), as shown in Figure 4a. The PeLEDs possess relatively low turn-on voltages of about 3.0~3.3 V, with CIE chromaticity diagram (CIE) coordinates at (~0.05, 0.53~0.56) under an operating voltage of 6.0 V (Figure 4b), and the EL spectra are presented in Figures S8–S11. Along with the increasing operating voltages, all of the PeLEDs present higher current density and luminescence, as depicted in Figure 4c, proving efficient charge carrier transport and exciton combination inside the devices. Thus, the devices with ***m*-SFX-*m*F**, ***p*-SFX-*m*F**, ***m*-SFX-*o*F**, and ***p*-SFX-*o*F** used as HTLs show the maximum luminance (*L*_max_) of 5182 cd∙m^−2^, 3444 cd∙m^−2^, 3152 cd∙m^−2^ and 4377 cd∙m^−2^, respectively. Analyzing the measured efficiency plots (Figure 4d), the devices based on *m*F-unit-functionalized HTMs (***m*-SFX-*m*F** and ***p*-SFX-*m*F**) present superior performance relative to the other two PeLEDs, with maximum current efficiencies (CE_max_) of 4.94 cd·A^−1^ and 4.87 cd·A^−1^, maximum power efficiencies (PE_max_) of 4.69 lm∙W^−1^ and 5.09 lm∙W^−1^ and maximum external quantum efficiencies (EQE_max_) of 3.15% and 3.16%, respectively. These results suggest that the *m*F-unit modification is an effective strategy to exploit SFX-based HTMs for PeLEDs. Especially for ***m*-SFX-*m*F**, the corresponding device shows remarkable performance among this group of PeLED evaluations, which is summarized in Table 3, demonstrating that the ***m*-SFX-*m*F** is an ideal HTM candidate for fully inorganic PeLEDs. We speculate that the shrunken band-gap of the *m*F-based molecules promotes the charge carrier injection and exciton combination inside of the PeLEDs.

## 4. Conclusions

In summary, we designed and synthesized four isomeric SFX-based HTMs through regulating the substitution of the fluorine atom on the aniline units and the positions of the aniline unit on the xanthene moiety. The synergistic position effects of the fluorination on the FMO levels and of the amination on the thermal properties make the molecules efficient and versatile HTMs, which can be thermally evaporated to deposit HTLs for both inverted PSCs and fully inorganic PeLEDs. In the evaluations of the PSC performance, the HTM ***p*-SFX-*o*F** exhibits an improved average PCE of 14.29% with a maximum value of 15.21%, benefiting from the low HOMO level and good HTL/perovskite interface contact. Meanwhile, the mF-unit-substituted HTM exhibits an EQE_max_ of 3.15% with an L_max_ of 5182 cd∙m^−2^ for the CsPb(Br/Cl)_3_-based PeLED, which is attributed to the perched HOMO level and shrunken band-gap of ***m*-SFX-*m*F** for facilitating charge carrier injection and then exciton combination. These results reveal, to a certain extent, the relationship between the integrated position effect and the optoelectronic performance, thereby laying the foundation for the design and exploitation of highly efficient SFX-based HTMs.

## Figures and Tables

**Table 1:** Photophysical and electrochemical properties of the four molecules.

Compound	*λ*_abs_ *^a^* [nm]	*λ*_PL_ *^b^* [nm]	*E*_g_ *^c^* [eV]	*E*_HOMO_ *^d^* [eV]	*T*_d_ [°C]
***m*-SFX-*m*F**	303 ± 1	423 ± 1	2.99	−5.24	395
***m*-SFX-*o*F**	304 ± 1	413 ± 1	3.06	−5.24	398
***p*-SFX-*m*F**	302 ± 1	423 ± 1	2.99	−5.23	403
***p*-SFX-*o*F**	303 ± 1	413 ± 1	3.05	−5.25	425

*^a^* UV–vis absorbance peak measured in toluene solution (10^−5^ M). *^b^* Photoluminescent peak measured in CH_2_Cl_2_ solution (10^−5^ M). *^c^* Optical gap determined from the edge of the absorption spectra. *^d^* The HOMO levels were calculated according to the formula −[5.1 eV ± *E*_ox_ (vs *E*_Fc/Fc+_)], Fc/Fc^+^: ferrocene/ferrocenium.

**Table 2:** Photovoltaic parameters of the PSCs based on dopant-free HTMs.

HTMs	*V*_oc_ [V]	*J*_sc_ [mA·cm^−2^]	*FF* [%]	PCE [%]	
				Average	Maximum
***m*-SFX-*m*F**	1.07 ± 0.01	17.72 ± 0.76	63.01 ± 9.56	11.68	13.82
***m*-SFX-*o*F**	1.14 ± 0.01	17.52 ± 1.21	63.64 ± 8.40	12.70	15.27
***p*-SFX-*m*F**	1.03 ± 0.03	17.64 ± 0.46	70.09 ± 4.65	12.59	13.52
***p*-SFX-*o*F**	1.13 ± 0.01	17.99 ± 0.62	72.63 ± 1.72	14.29	15.21

**Table 3:** Summary of the electroluminescent performances of ***m*-SFX-*o*F**-, ***m*-SFX-*m*F**-, ***p*-SFX-*o*F**- and ***p*-SFX-*m*F**-based PeLEDs.

Device	*V ^a^* [V]	*CE ^b^* [cd·A^−1^]	*PE ^b^* [lm·W^−1^]	*EQE ^b^* [%]	*L*_max_ [cd·m^−2^]	*CIE ^c^*
***m*** **-SFX-*m*F**	3.3, 3.8	4.94, 2.79	4.69, 1.82	3.15, 1.69	5182	(0.05, 0.53)
***m*** **-SFX-*o*F**	3.3, 4.0	3.81, 2.19	3.63, 1.29	2.45, 1.30	3444	(0.05, 0.55)
***p*** **-SFX-*m*F**	3.0, 4.7	4.87, 2.25	5.09, 1.27	3.16, 1.31	3152	(0.05, 0.55)
***p*** **-SFX-oF**	3.0, 4.1	4.24, 2.37	4.44, 1.45	2.71, 1.43	4377	(0.05, 0.56)

*^a^* Operating voltage at luminance of onset and 100 cd·m^−2^; *^b^* Values of *CE*, *PE* and *EQE* at their maximum and 100 mA cm^−2^; *^c^* Recorded under an operating voltage of 6.0 V.

## Data Availability

The data presented in this study are available on request from the corresponding author.

## Supplementary Materials

The following supporting information can be downloaded at https://www.mdpi.com/article/10.3390/nano14121044/s1, Figure S1: Optimized molecular configurations of *m*-SFX*-oF*, *p*-SFX*-oF*, *m*-SFX*-mF*, and *p*-SF *-mF*; Figure S2: Fluorescent emission spectra of *m*-SFX*-oF*, *p*-SFX*-oF*, *m*-SFX*-mF*, and *p*-SFX *-mF* in a dichloromethane solution; Figure S3: Thermogravimetric diagram of *m*-SFX*-oF*, *p*-SFX*-oF*, *m*-SFX*-mF*, and *p*-SFX *-mF*; Figure S4: *J-V* curves of *m*-SFX-*m*F at 10−30 nm HTLs; Figure S5: *J-V* curves of *m*-SFX-*o*F at 6−20 nm HTLs; Figure S6: *J-V* curves of *p*-SFX-*m*F at 10−30 nm HTMs; Figure S7: *J-V* curves of *p*-SFX-*o*F at 10−30 nm HTLs; Table S1: PSC performances of the four HTMs with different thicknesses; Figure S8: The ^1^H NMR spectra of DPA-*o*F (DMSO-*d_6_*, 400 MHz); Figure S9: The ^1^H NMR spectra of DPA-*m*F (DMSO-*d_6_*, 400 MHz); Figure S10: The ^1^H NMR spectra of *m*-SFX-*o*F (DMSO-*d_6_*, 400 MHz); Figure S11: The ^13^C NMR spectra of *m*-SFX-*o*F (DMSO-*d_6_*, 400 MHz); Figure S12: The ^1^H NMR spectra of *p*-SFX-*o*F (DMSO-*d_6_*, 400 MHz); Figure S13: The ^13^C NMR spectra of *p*-SFX-*o*F (DMSO-*d_6_*, 400 MHz); Figure S14: The ^1^H NMR spectra of *m*-SFX-*m*F (DMSO-*d_6_*, 400 MHz); Figure S15: The ^13^C NMR spectra of *m*-SFX-*m*F (DMSO-*d_6_*, 400 MHz); Figure S16: The ^1^H NMR spectra of *p*-SFX-*m*F (DMSO-*d_6_*, 400 MHz); Figure S17: The ^13^C NMR spectra of *p*-SFX-*m*F (DMSO-*d_6_*, 400 MHz). Figure S18: The ^1^H NMR spectra of *m*-SFX-*m*F (DMSO-*d*6, 400 MHz); Figure S19: The ^13^C NMR spectra of *m*-SFX-*m*F (DMSO-*d*6, 400 MHz); Figure S20: The ^1^H NMR spectra of *p*-SFX-*m*F (DMSO-*d*6, 400 MHz); Figure S21: The ^13^C NMR spectra of *p*-SFX-*m*F (DMSO-*d*6, 400 MHz).
