# Supplementary material for: Evaluating Fluorinated-Aniline Units with Functionalized Spiro[Fluorene-9,9′-Xanthene] as Hole-Transporting Materials in Perovskite Solar Cells and Light-Emitting Diodes
Source: Nanomaterials (Basel). 2024 Jun 18;14(12):1044. doi: 10.3390/nano14121044 (PMC11206255; doi:10.3390/nano14121044)
Supplement: Supplementary file 1 [file nanomaterials-14-01044-s001.zip › nanomaterials-3052213-supplementary.pdf]

## *Supporting Information*

# Evaluating Fluorinated-Aniline Units with Functionalized Spiro[Fluorene-9,9′-Xanthene] as Hole-Transporting Materials in Perovskite Solar Cells and Light-Emitting Diodes

**Kuo Liu [1,†], Liang Sun [2,†], Qing-Lin Liu [1,†], Bao-Yi Ren [1,*], Run-Da Guo [2,*], Lei Wang [2], Ya-Guang Sun [1] and You-Sheng Wang [3,*]**

1 Key Laboratory of Inorganic Molecule-Based Chemistry of Liaoning Province, College of Science, Shenyang University of Chemical Technology, Shenyang 110142, China; 15233487432@163.com (K.L.); lql1347585318@163.com (Q.-L.L.); sunyaguang@syuct.edu.cn (Y.-G.S.)
2 Wuhan National Laboratory for Optoelectronics, Huazhong University of Science and Technology, Wuhan 430074, China; lsiuang@hotmail.com (L.S.); wanglei@mail.hust.edu.cn (L.W.)
3 Institute of New Energy Technology, College of Physics & Optoelectronic Engineering, Jinan University, Guangzhou 510632, China
† These authors contributed equally to this work.
* Correspondence: renbaoyi@syuct.edu.cn (B.-Y.R.); runda_guo@hust.edu.cn (R.-D.G.); wangys0120@jnu.edu.cn (Y.-S.W.) Tel.: +86-024-8938-3302 (B.-Y.R.)

## *1. PSCs Device Characterization*

Current density–voltage (J–V) characteristics of the solar cells were measured using a Keithley 2400 source meter. The illumination was provided by a Newport Oriel 92192 solar simulator with an AM1.5 G filter, operating at 100 mW cm$^{-2}$, which was calibrated by a standard silicon solar cell from Newport. Both forward and backward scans were performed, and the scan speed was fixed at 0.15 V s$^{-1}$. The crystal structure was characterized by Bruker D8 Advance X-ray diffractometer (XRD) with Cu $K_{\alpha}$ radiation operated at 40 kV and 40 mA. The transient photovoltage/current decay measurements were performed on an electrochemical workstation (ZAHNER, Germany). PL was measured by the FLS980 (Edinburgh Instruments Ltd.) with an excitation at 470 nm. The EQE was taken using a QE-R instrument from Enlitech.

## *2. Materials and methods*

***2.1 Synthesis:*** All solvents and chemicals, unless otherwise indicated, were purchased from commercial suppliers without further purification. All reactions were monitored by thin layer chromatographic analysis on a pre-coated silica gel plate, which was visualized by a UV lamp at 254 or 365 nm. Flash column chromatography was performed on glass column of silica gel (200–300 mesh) and solvent ratios were expressed in volume to volume.

***2.1.1 Synthesis of m*F-unit:** 4-bromo-2-fluoro-1-methoxybenzene (5.24ml, 40.60mmol), 4-methoxyaniline (5.00 g, 40.60 mmol), tri(tert-butyl)phosphine tetrafluoroborate (353.38 mg, 1.22 mmol), tris(dibenzylideneacetone)dipalladium (743.56 mg, 811.98 μmol) and sodium tert-butylalcohol (7.8 g, 81.20 mmol) were added into 250 mL three-mouth flask successively under the protection of argon, and then 68 mL drying toluene was added. After sealing, the reaction was reflowing at 110 °C for 4 h, and the reaction process was monitored by thin-layer chromatography. When the reaction cooled to room temperature, the reaction liquid was washed with saturated sodium chloride solution, and then the organic phase was extracted with methylene chloride. The

collected organic phase was dried by anhydrous magnesium sulfate, filtered and spun dry, and purified by column chromatography (DCM: PE = 1:1.5) to obtain pure product as yellow transparent oil liquid (8.03 g, 80.04%). $^{1}$H NMR (500 MHz, DMSO-*d$_6$*) δ: 7.29 (s, 1H), 7.06 (tt, J = 6.3, 3.0 Hz, 1H), 6.87 (m, 1H), 6.84 – 6.79 (m, 4H), 6.69 (ddd, J = 8.9, 2.8, 1.3 Hz, 1H), 3.72 (s, 3H), 3.65 (s, 3H).

***2.1.2 Synthesis of o*F-unit*:*** *oF*-unit was synthesized through same procedure with *m*F-unit. 4-bromo-3-fluoro-1-methoxybenzene was used instead of 4-bromo-2-fluoro-1-methoxybenzene. The pure product was purified by column chromatography (EA: PE = 1:3.5) as light-yellow transparent oil liquid (7.86 g, 79.35% yield). $^{1}$H NMR (500 MHz, DMSO-*d$_6$*) δ: 7.73 (s, 1H), 6.97 (tt, J = 6.3, 3.0 Hz, 3H), 6.86 – 6.79 (m, 2H), 6.74 (dd, J = 13.7, 2.6 Hz, 1H), 6.67 (ddd, J = 8.9, 2.8, 1.3 Hz, 1H), 3.73 (s, 3H), 3.68 (s, 3H)

***2.1.3 Synthesis of m*-SFX-*o*F:** To a three-mouth round-bottomed flask, *oF*-unit (1.23 g, 4.97 mmol), 2,2',7,7'-tetrabromospiro[fluorene-9,9'-xanthene] (715.00 mg, 1.104 mmol), tri(tert-butyl)phosphine tetrafluoroborate (51.25 mg, 0.177 mmol), tris(dibenzylideneacetone)dipalladium (101.1 mg, 0.110 mmol) and sodium *tert*-butoxide (530.47 mg, 5.52 mmol) were added dissolved in 180 mL anhydrous toluene, and heated at 120 °C for 12h under $N_2$ atmosphere, and the reaction process was monitored by thin-layer chromatography. When the reaction was cooled to room temperature, washed with saturated sodium chloride solution, and then the organic phase was extracted with methylene chloride. The collected organic phase was dried by anhydrous magnesium sulfate, filtered and spun dry, and purified by column chromatography (DCM: PE = 1:2) to obtain pure product as white solid (1.32 g, 91%). $^{1}$H NMR (400 MHz, DMSO-*d$_6$*) δ: 7.42 (d, J = 8.3 Hz, 2H), 7.06 – 6.90 (m, 6H), 6.90 – 6.73 (m, 18H), 6.72 – 6.45 (m, 12H), 6.10 (d, J = 2.7 Hz, 2H), 3.73 (dd, J = 23.5, 18.3 Hz, 24H). $^{13}$C NMR (101 MHz, DMSO-*d$_6$*) δ: 160.22, 160.01, 158.66, 158.55, 158.37, 158.27, 157.74, 157.54, 155.97, 155.08, 154.59, 147.42, 145.89, 143.24, 140.44, 139.75, 131.85, 130.59, 130.29, 126.90, 126.79, 126.73, 126.62, 125.71, 125.40, 123.18, 120.98, 120.33, 118.58, 118.25, 117.58, 115.11, 114.93, 114.56, 111.61, 111.51, 103.53, 103.47, 103.30, 103.23, 56.18, 55.65,54.52. HRMS for $C_{81}H_{64}F_4N_4O_9$: $[M + H]^+$ calcd. 1313.4683, found 1313.4631.

***2.1.4 Synthesis of p*-SFX-*o*F:** *p*-SFX-*o*F was synthesized through same procedure with *m*-SFX-*o*F. 2,3',6',7-tetrabromospiro[fluorene-9,9'-xanthene] was used instead of 2,2',7,7'-tetrabromospiro [fluorene-9,9'-xanthene]. The pure product was purified by column chromatography as white solid (1.28 g, 89% yield). $^{1}$H NMR (400 MHz, DMSO-*d$_6$*) δ: 7.51 (d, J = 8.3 Hz, 2H), 7.19 (t, J = 9.1 Hz, 2H), 7.12 – 7.00 (m, 6H), 6.96 – 6.75 (m, 18H), 6.75 – 6.68 (m, 2H), 6.60 (dd, J = 8.3, 2.2 Hz, 2H), 6.47 (d, J = 2.2 Hz, 2H), 6.28 – 6.18 (m, 4H), 6.14 (d, J = 2.0 Hz, 2H), 3.75 (dd, J = 16.8, 6.2 Hz, 24H). $^{13}$C NMR (101 MHz, DMSO-*d$_6$*) δ: 160.44, 160.03, 159.06, 158.95, 158.50, 158.39, 157.97, 157.56, 156.69, 155.87, 155.15, 151.66, 148.49, 147.40, 139.88, 139.06, 132.25, 131.22, 130.55, 127.94, 126.85, 126.75, 126.25, 126.13, 125.29, 120.42, 118.38, 116.72, 115.38, 115.07, 113.04, 111.79, 111.60, 104.21, 103.56, 103.33, 103.26, 56.20, 56.18, 55.69, 55.66, 53.22, 28.94. HRMS for $C_{81}H_{64}F_4N_4O_9$: $[M + H]^+$ calcd. 1313.4683, found 1313.4623.

***2.1.5 Synthesis of m*-SFX-*m*F:** *m*-SFX-*m*F was synthesized through same procedure with *m*-SFX-*o*F. *oF*-unit was used instead of *oF*-unit. The pure product was purified by column chromatography as white solid (1.06 g, 84% yield). $^{1}$H NMR (400 MHz, DMSO-*d$_6$*) δ: 7.35 (d, *J* = 8.3 Hz, 2H), 7.02 (t, *J* = 9.1 Hz, 2H), 6.95 (t, *J* = 9.4 Hz, 4H), 6.89 – 6.58 (m, 26H), 6.53 (dd, *J* = 8.3, 2.2 Hz, 2H), 6.44 (d, *J* = 2.2 Hz, 2H), 5.99 (d, *J* = 2.8 Hz, 2H), 3.82 – 3.64 (m, 24H). $^{13}$C NMR (101 MHz, DMSO-*d$_6$*) δ: 156.43, 156.00, 154.95, 153.25, 150.82, 147.47, 146.52, 143.48, 143.37, 143.24,

142.67, 142.56, 141.72, 141.64, 141.06, 140.98, 140.21, 139.98, 132.62, 127.03, 126.12, 125.51, 124.08, 121.59, 121.29, 120.83, 120.08, 118.29, 118.08, 117.21, 115.38, 115.26, 115.07, 112.06, 111.86, 110.50, 110.30, 56.67, 56.62, 55.66, 55.63, 54.29. HRMS for $C_{81}H_{64}F_4N_4O_9$: $[M + H]^+$ calcd. 1313.4683, found 1313.4639.

***2.1.6 Synthesis of p*-SFX-*m*F:** *p*-SFX-*m*F was synthesized through same procedure with *m*-SFX-*m*F. 2,3',6',7-tetrabromospiro[fluorene-9,9'-xanthene] was used instead of 2,2',7,7'-tetrabromospiro [fluorene-9,9'-xanthene]. The pure product was purified by column chromatography as white solid (0.901 g, 89% yield). $^1$H NMR (400 MHz, DMSO-$d_6$) δ: 7.51 (d, $J$ = 8.3 Hz, 2H), 7.19 (t, $J$ = 9.1 Hz, 2H), 7.12 – 7.00 (m, 6H), 6.97 – 6.76 (m, 18H), 6.76 – 6.67 (m, 2H), 6.60 (dd, $J$ = 8.2, 2.3 Hz, 2H), 6.47 (d, $J$ = 2.2 Hz, 2H), 6.28 – 6.17 (m, 4H), 6.14 (d, $J$ = 2.0 Hz, 2H), 3.75 (dd, $J$ = 16.8, 6.2 Hz, 24H). $^{13}$C NMR (101 MHz, DMSO-$d_6$) δ: 160.44, 160.03, 159.05, 158.95, 158.49, 158.39, 157.97, 157.56, 156.69, 155.87, 155.15, 151.66, 148.50, 147.40, 139.87, 139.05, 132.25, 131.22, 130.55, 127.93, 126.85, 126.75, 126.25, 126.13, 125.29, 120.42, 118.38, 116.72, 115.37, 115.07, 113.04, 111.78, 111.61, 104.20, 103.56, 103.49, 103.32, 103.26, 56.20, 56.18, 55.69, 55.66, 53.22. HRMS for $C_{81}H_{64}F_4N_4O_9$: $[M + H]^+$ calcd. 1313.4683, found 1313.4626.

## *3. Supporting figures*

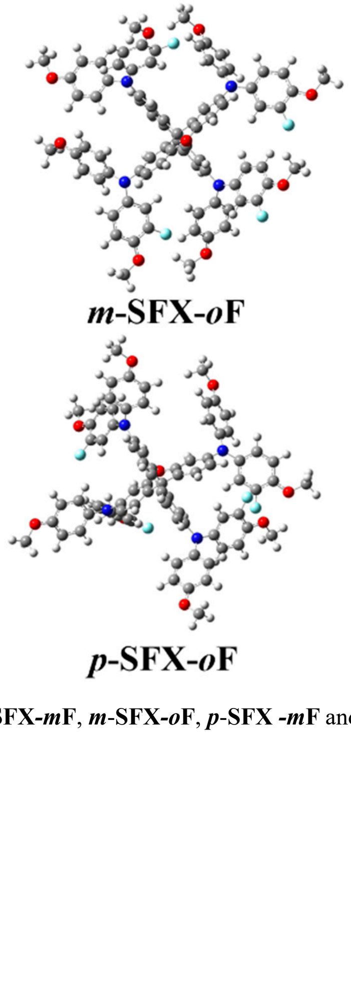


**Fig. S1** Optimized molecular configurations of ***m*-SFX-*m*F**, ***m*-SFX-*o*F**, ***p*-SFX -*m*F** and ***p*-SFX-*o*F**.

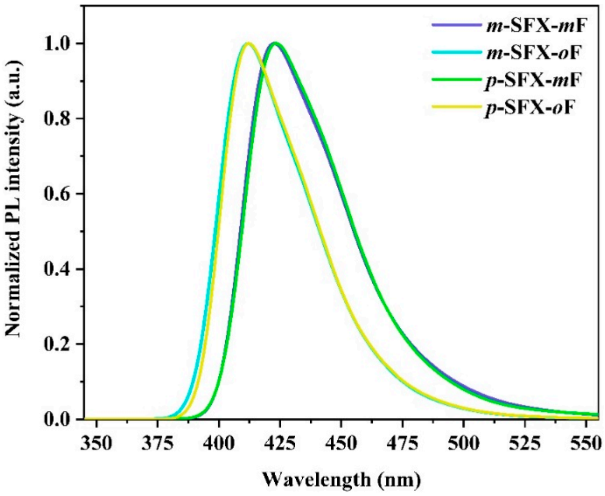


**Fig. S2** Fluorescent emission spectra of ***m*-SFX-*m*F**, ***m*-SFX-*o*F**, ***p*-SFX -*m*F** and ***p*-SFX-*o*F** in dichloromethane solution.

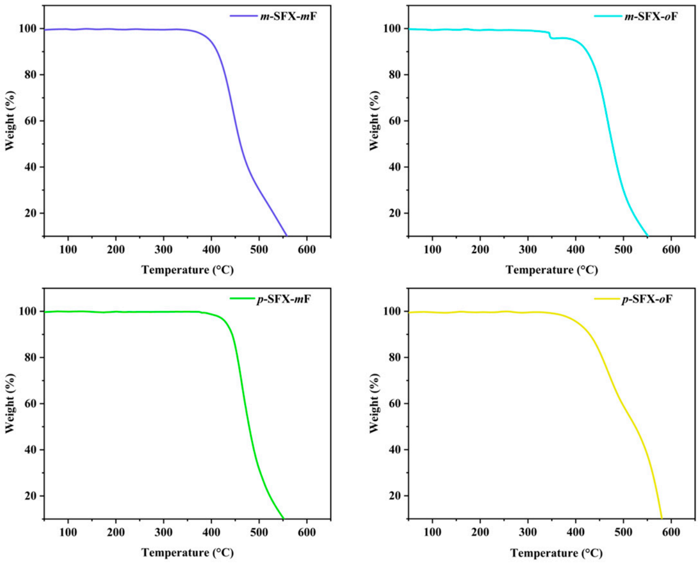


**Fig. S3** Thermogravimetric diagram of ***m*-SFX-*m*F**, ***m*-SFX-*o*F**, ***p*-SFX -*m*F** and ***p*-SFX-*o*F**.

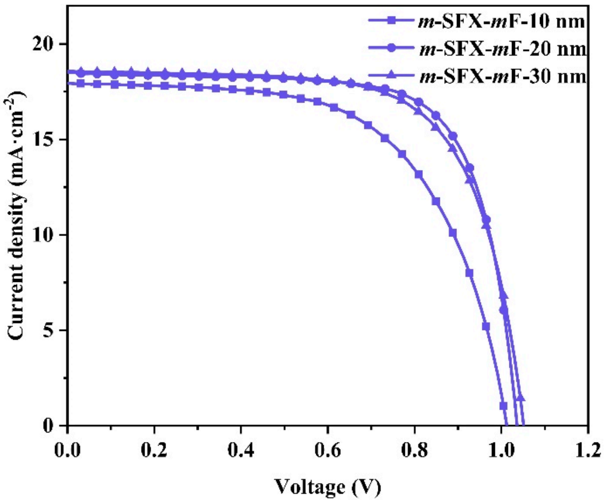


**Fig. S4** *J-V* curves of ***m*-SFX-*m*F** at 10−30nm HTLs.

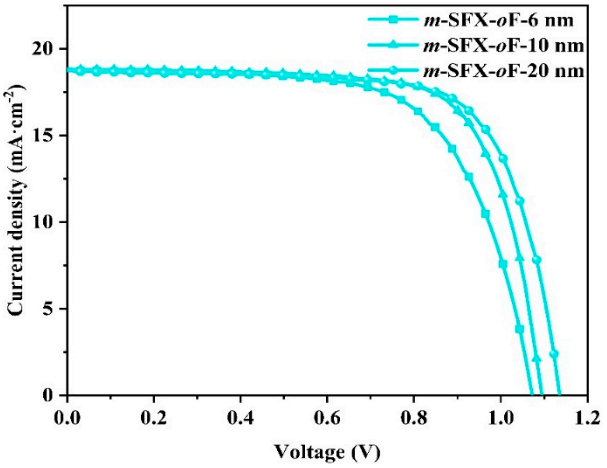


**Fig. S5** *J-V* curves of ***m*-SFX-*o*F** at 6−20nm HTLs.

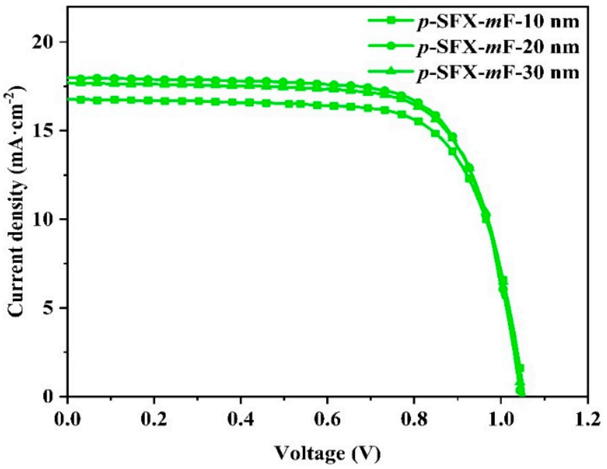


**Fig. S6** *J-V* curves of ***p*-SFX-*m*F** at 10−30nm HTMs.

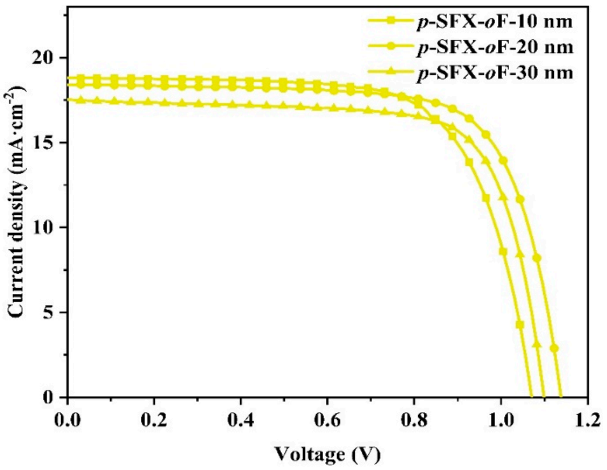


**Fig. S7** *J-V* curves of ***p*-SFX-*o*F** at 10−30nm HTLs.

**Table S1** PSC performances of the four HTMs with different thicknesses.

| HTM | *Thicknesses* (nm) | $V_{oc}$ (V) | $J_{sc}$ (mA·cm$^{-2}$) | *FF* (%) | *PCE* (%) |
|---|---|---|---|---|---|
| ***m*-SFX-*m*F** | 10 | 1.01 | 17.90 | 61 | 11.04 |
| | 20 | 1.08 | 18.48 | 73 | 13.82 |
| | 30 | 1.04 | 18.55 | 69 | 13.35 |
| ***m*-SFX-*o*F** | 6 | 1.07 | 18.78 | 66 | 13.26 |
| | 10 | 1.08 | 18.78 | 73 | 14.89 |
| | 20 | 1.15 | 18.73 | 72 | 15.27 |
| ***p*-SFX-*m*F** | 10 | 1.04 | 16.73 | 72 | 12.63 |
| | 20 | 1.06 | 18.10 | 75 | 13.52 |
| | 30 | 1.04 | 17.66 | 72 | 13.33 |
| ***p*-SFX-*o*F** | 10 | 1.07 | 18.79 | 70 | 13.98 |
| | 20 | 1.14 | 18.61 | 74 | 15.21 |
| | 30 | 1.10 | 17.49 | 74 | 14.12 |

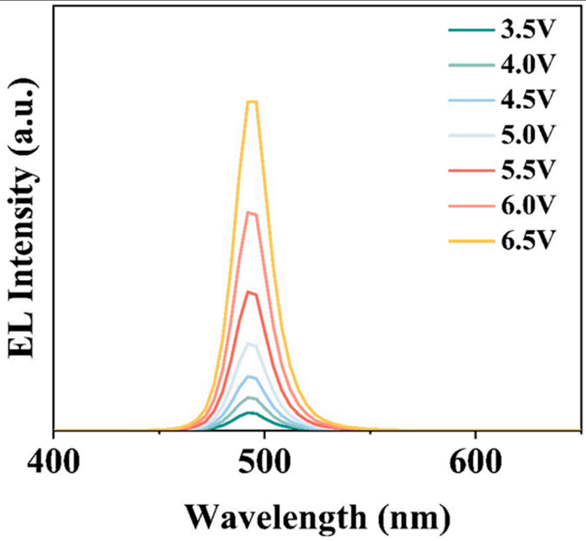


**Fig. S8** The EL spectra of PeLED using the ***m*-SFX-*m*F** as HTL under different bias voltages

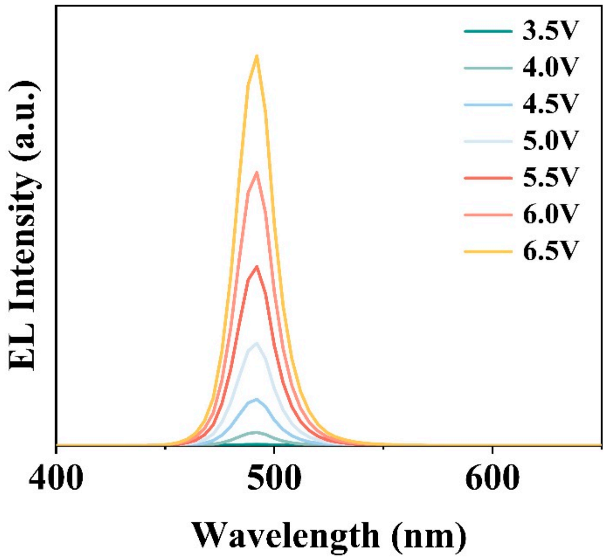


**Fig. S9** The EL spectra of PeLED using the ***m*-SFX-*o*F** as HTL under different bias voltages

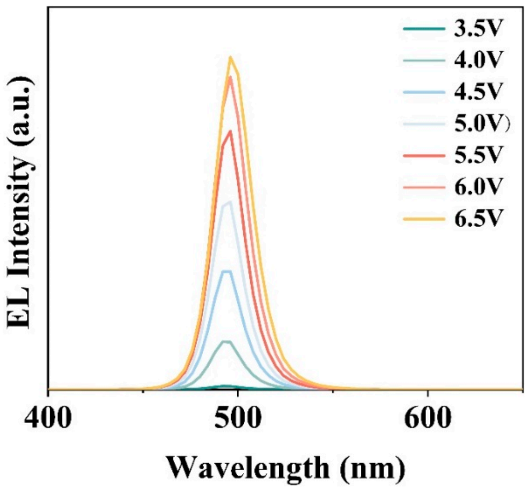


**Fig. S10** The EL spectra of PeLED using the ***p*-SFX-*m*F** as HTL under different bias voltages

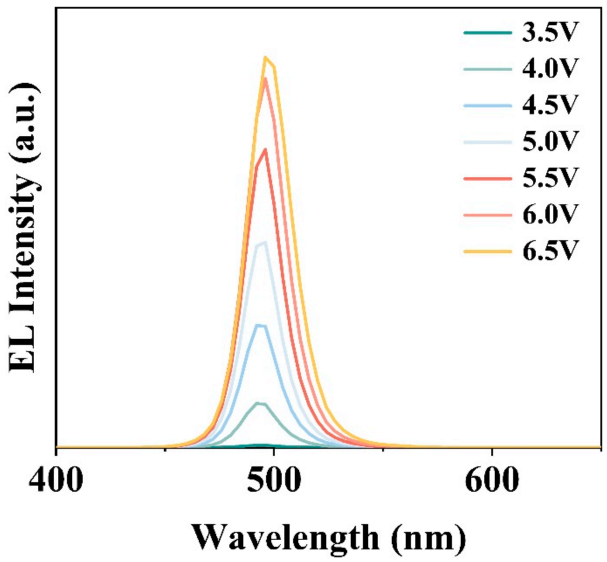


**Fig. S11** The EL spectra of PeLED using the ***p*-SFX-*o*F** as HTL under different bias voltages

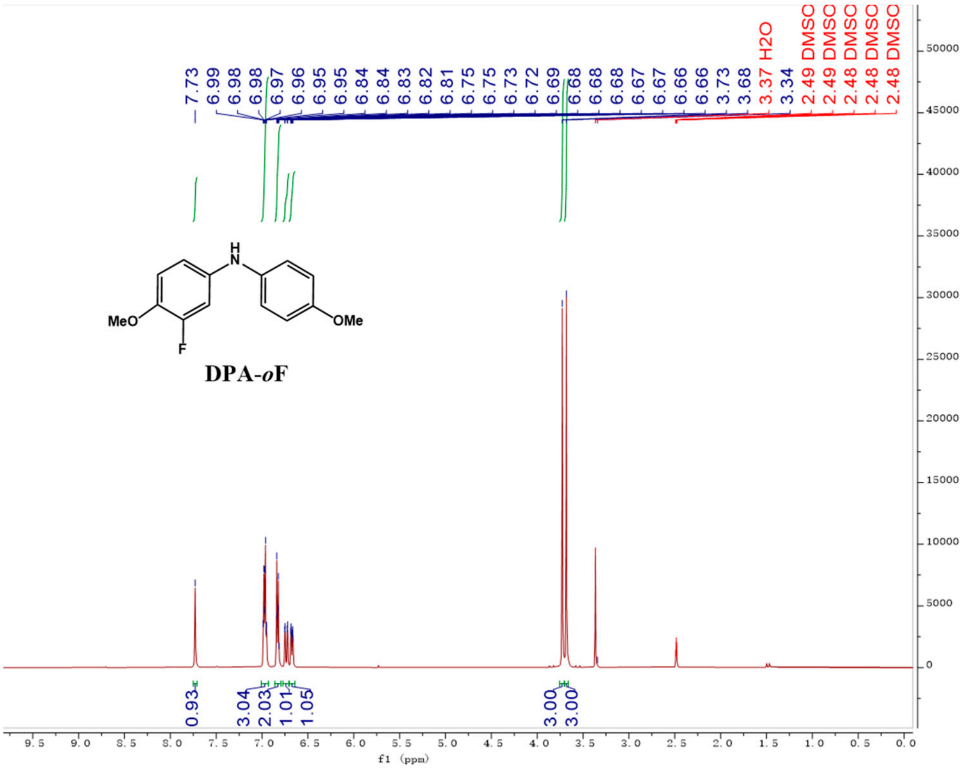


**Fig. S12** The $^{1}$H NMR spectra of DPA-*o*F (DMSO-$d_6$, 400 MHz)

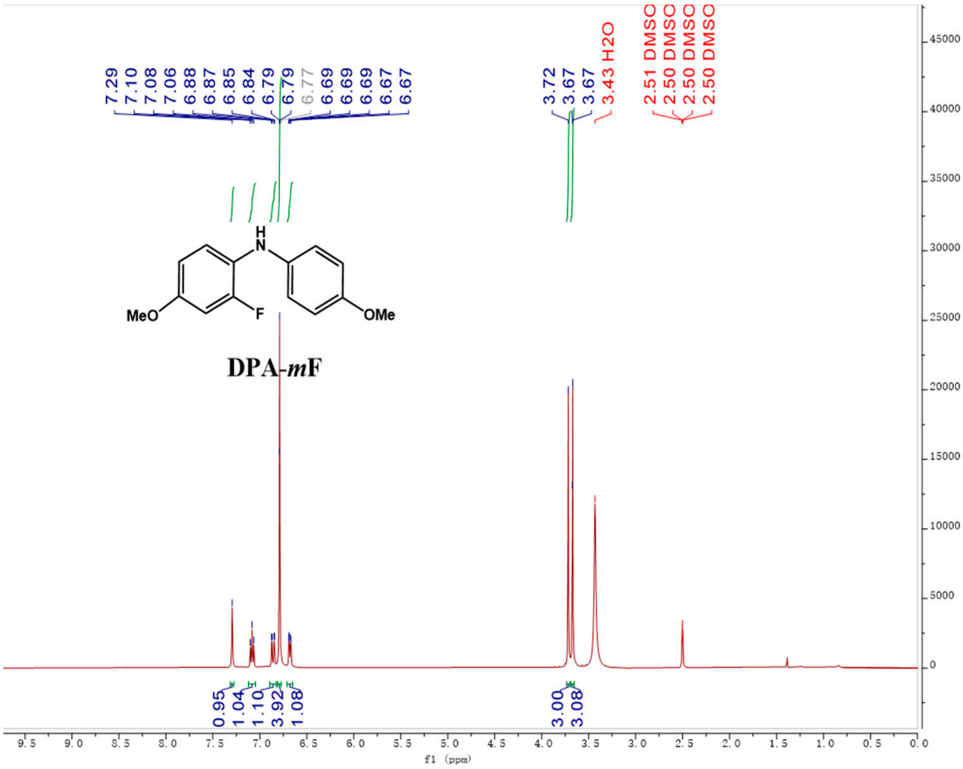


**Fig. S13** The $^{1}$H NMR spectra of DPA-*m*F (DMSO-$d_6$, 400 MHz)

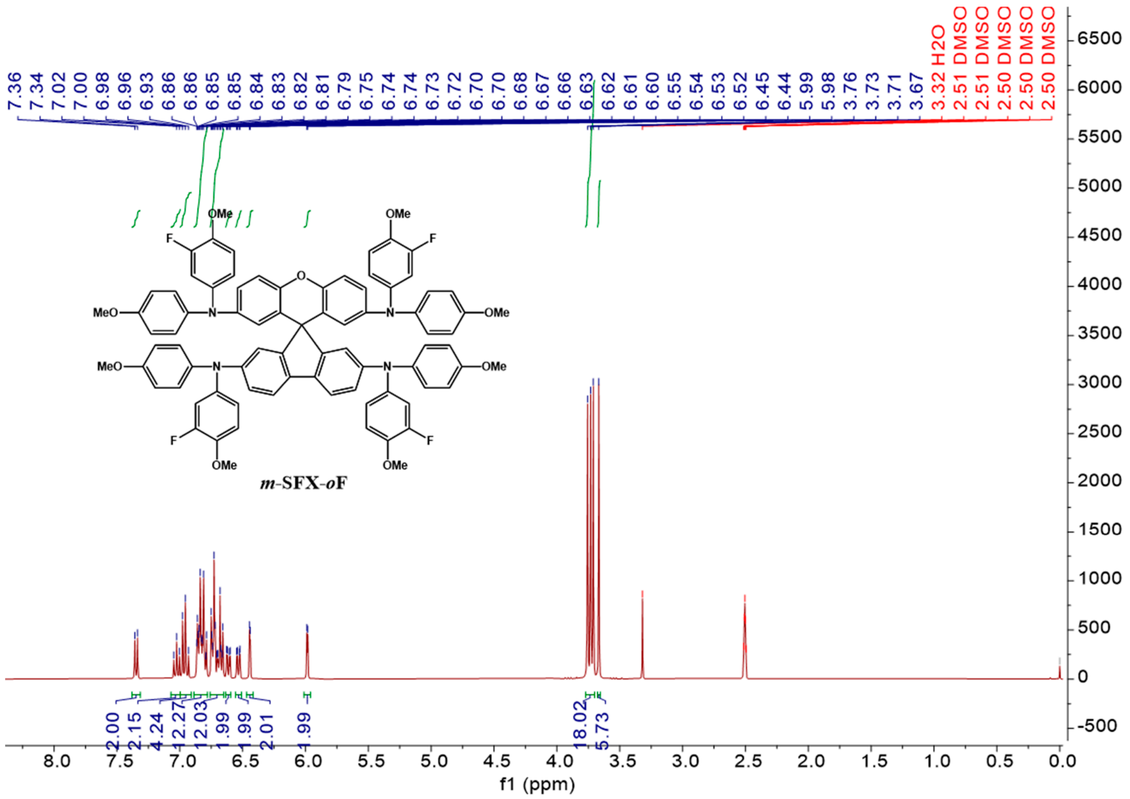

**Fig. S14** The $^{1}$H NMR spectra of *m*-SFX-*o*F (DMSO-$d_6$, 400 MHz)

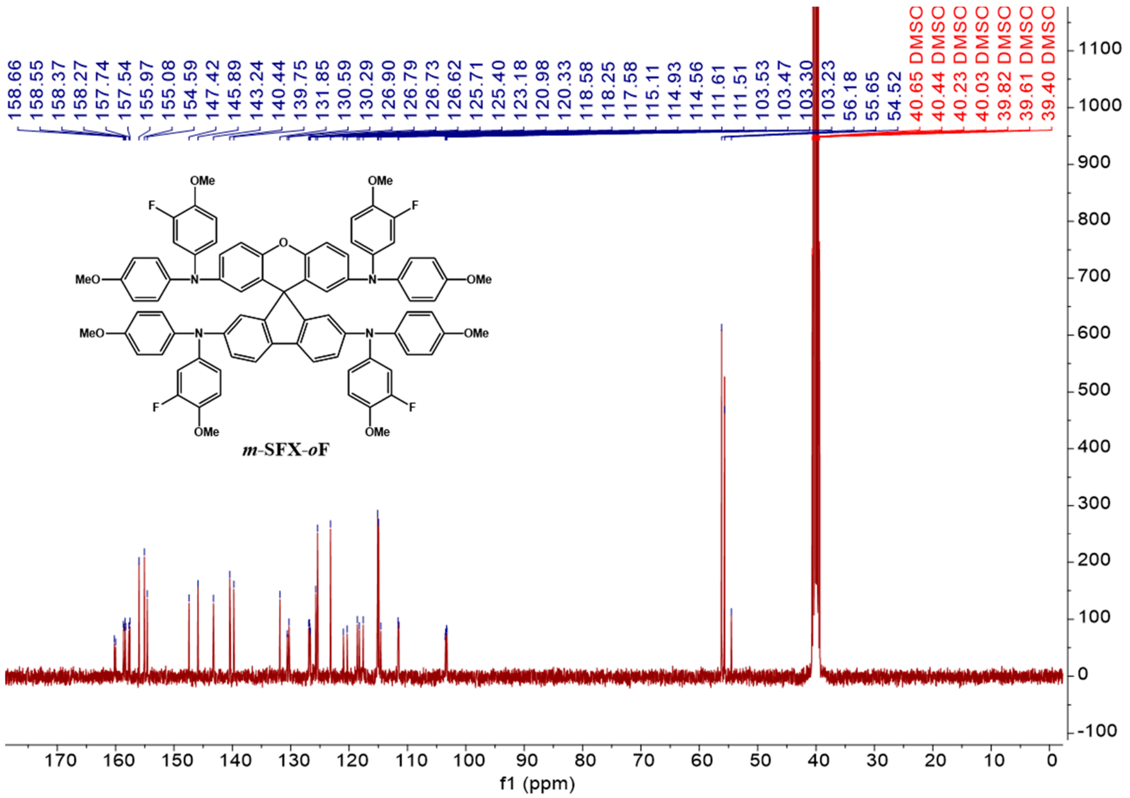

**Fig. S15** The $^{13}$C NMR spectra of *m*-SFX-*o*F (DMSO-$d_6$, 400 MHz)

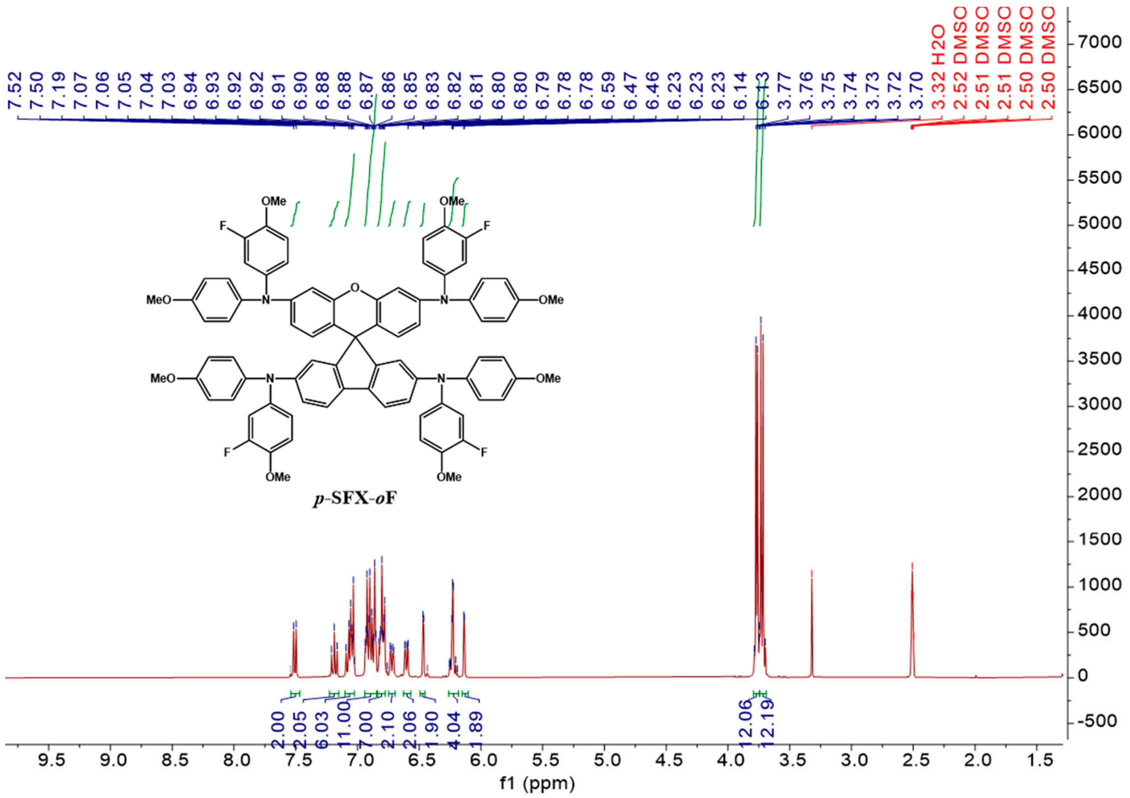

**Fig. S16** The $^{1}$H NMR spectra of *p*-SFX-*o*F (DMSO-$d_6$, 400 MHz)

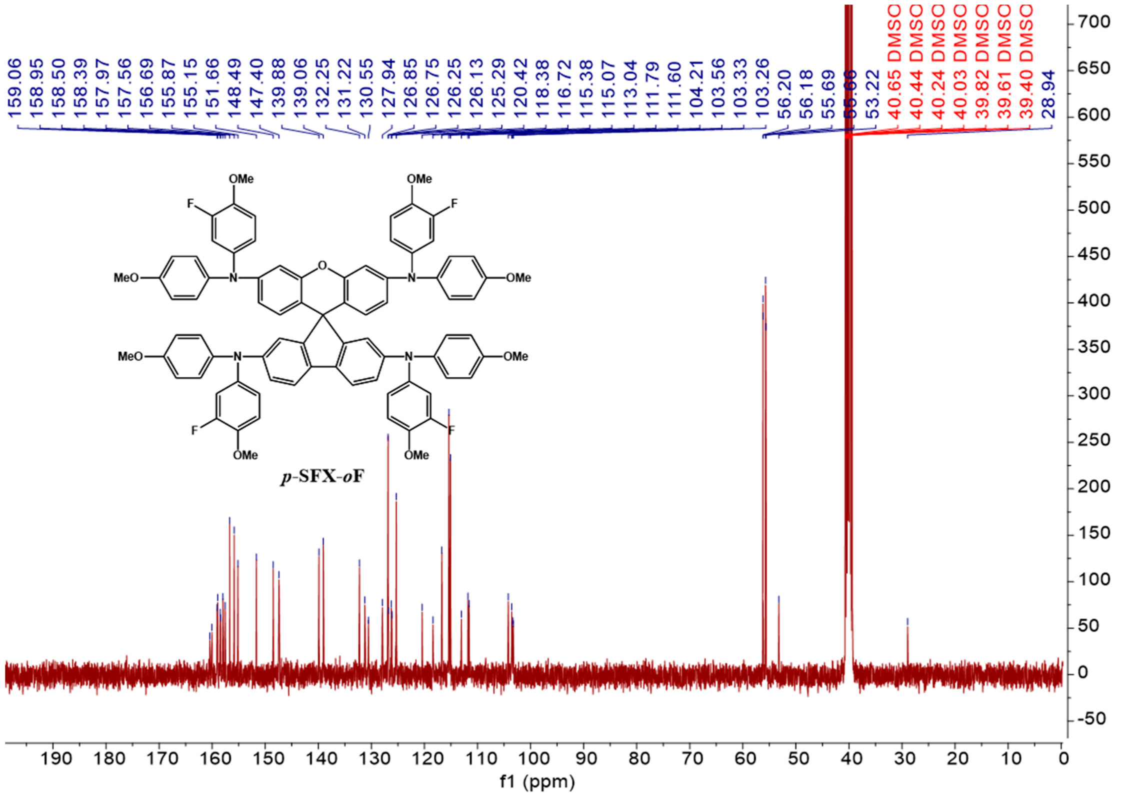

**Fig. S17** The $^{13}$C NMR spectra of *p*-SFX-*o*F (DMSO-$d_6$, 400 MHz)

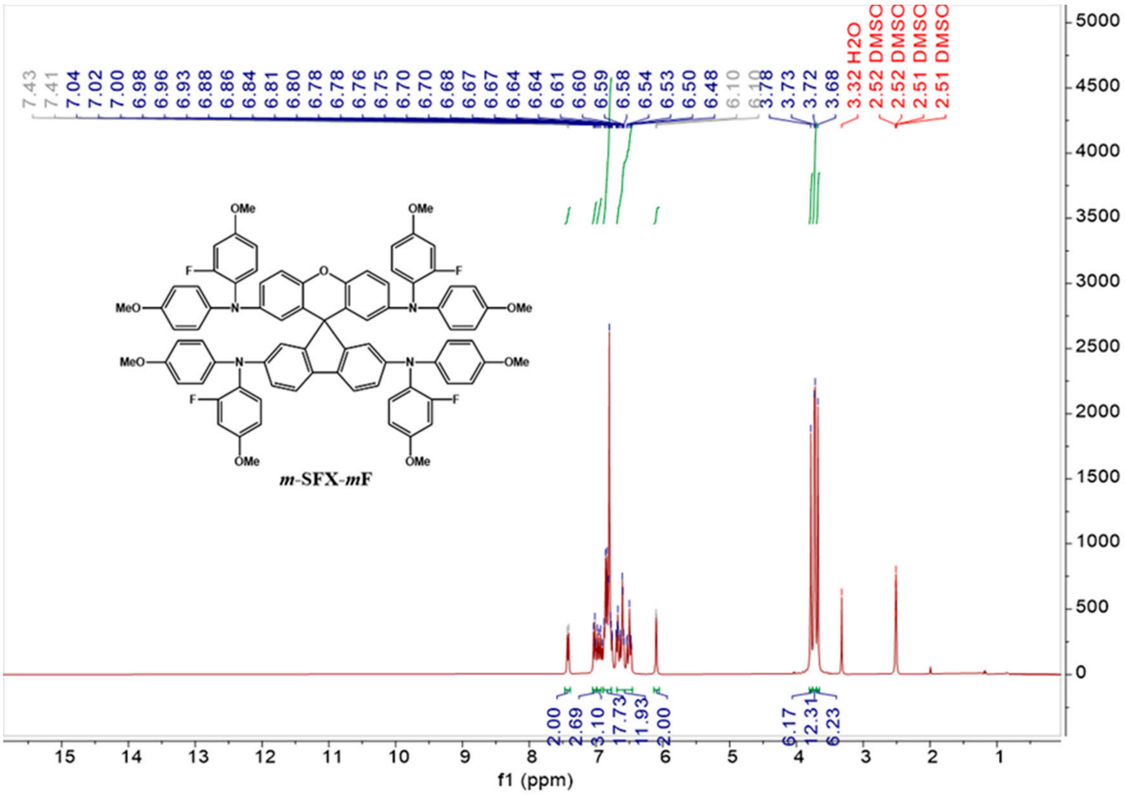

**Fig. S18** The $^{1}$H NMR spectra of *m*-SFX-*m*F (DMSO-$d_6$, 400 MHz)

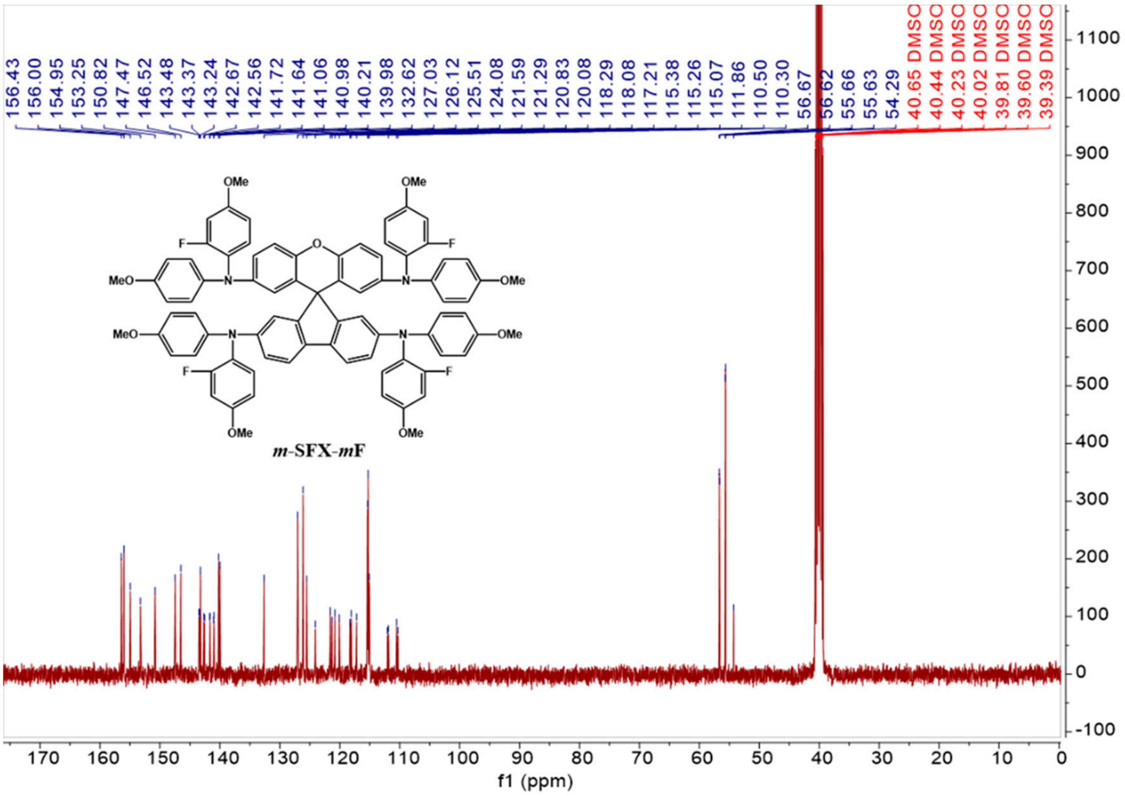

**Fig. S19** The $^{13}$C NMR spectra of *m*-SFX-*m*F (DMSO-$d_6$, 400 MHz)

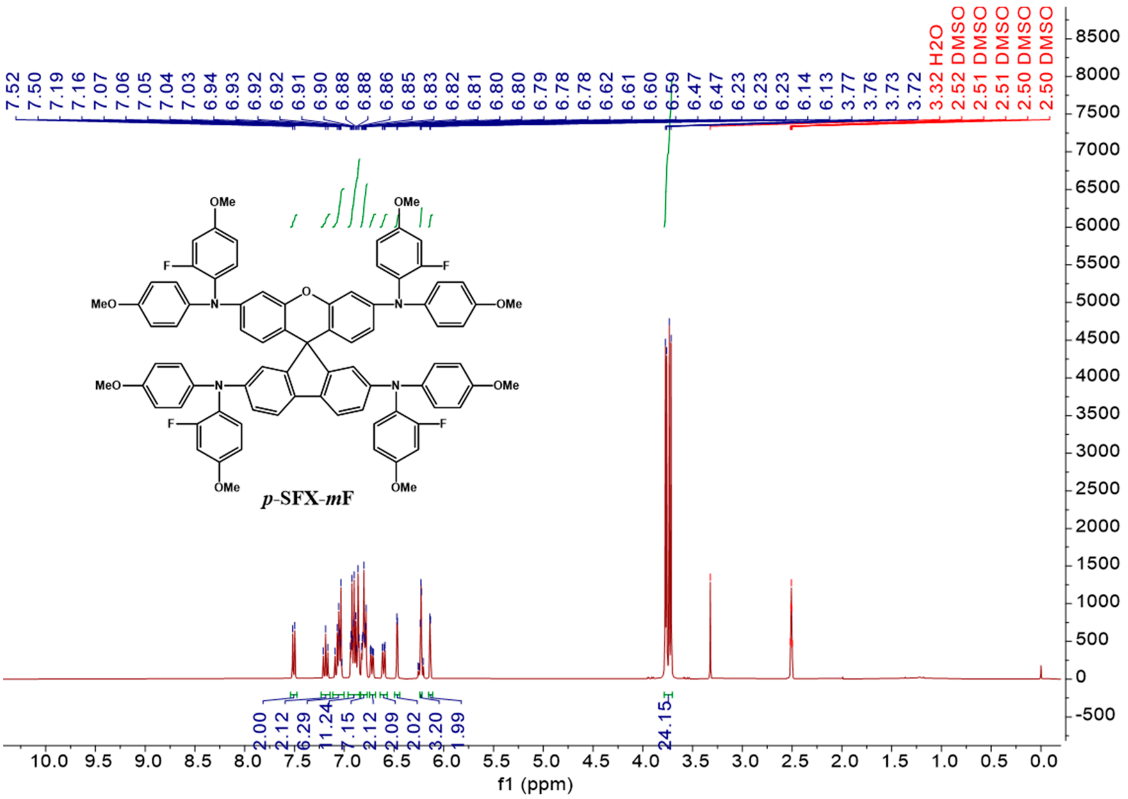

**Fig. S20** The $^{1}$H NMR spectra of *p*-SFX-*m*F (DMSO-$d_6$, 400 MHz)

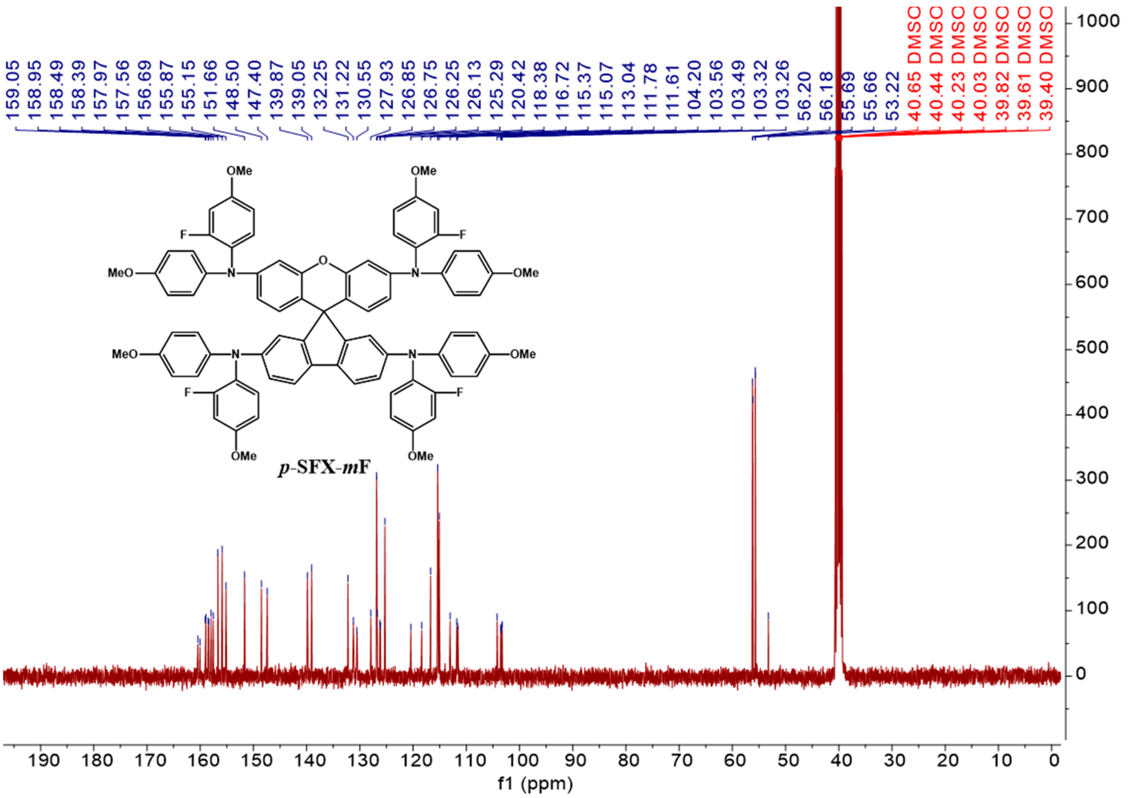

**Fig. S21** The $^{13}$C NMR spectra of *p*-SFX-*m*F (DMSO-$d_6$, 400 MHz)
